# Evidence summaries: the evolution of a rapid review approach

**DOI:** 10.1186/2046-4053-1-10

**Published:** 2012-02-10

**Authors:** Sara Khangura, Kristin Konnyu, Rob Cushman, Jeremy Grimshaw, David Moher

**Affiliations:** 1 Clinical Epidemiology Program, Ottawa Hospital Research Institute, 501 Smyth Road, Box 208, Ottawa, Ontario, K1H 8L6, Canada; 2 Champlain Local Health Integration Network, 1900 City Park Drive, Suite 204, Ottawa, Ontario, K1J 1A3, Canada; 3 Epidemiology and Community Medicine, University of Ottawa, 451 Smyth Road, Room 3105, Ottawa, Ontario, K1H 8M5, Canada

## Abstract

**Background:**

Rapid reviews have emerged as a streamlined approach to synthesizing evidence - typically for informing emergent decisions faced by decision makers in health care settings. Although there is growing use of rapid review 'methods', and proliferation of rapid review products, there is a dearth of published literature on rapid review methodology. This paper outlines our experience with rapidly producing, publishing and disseminating evidence summaries in the context of our Knowledge to Action (KTA) research program.

**Methods:**

The KTA research program is a two-year project designed to develop and assess the impact of a regional knowledge infrastructure that supports evidence-informed decision making by regional managers and stakeholders. As part of this program, we have developed evidence summaries - our form of rapid review - which have come to be a flagship component of this project. Our eight-step approach for producing evidence summaries has been developed iteratively, based on evidence (where available), experience and knowledge user feedback. The aim of our evidence summary approach is to deliver quality evidence that is both timely and user-friendly.

**Results:**

From November 2009 to March 2011 we have produced 11 evidence summaries on a diverse range of questions identified by our knowledge users. Topic areas have included questions of clinical effectiveness to questions on health systems and/or health services. Knowledge users have reported evidence summaries to be of high value in informing their decisions and initiatives. We continue to experiment with incorporating more of the established methods of systematic reviews, while maintaining our capacity to deliver a final product in a timely manner.

**Conclusions:**

The evolution of the KTA rapid review evidence summaries has been a positive one. We have developed an approach that appears to be addressing a need by knowledge users for timely, user-friendly, and trustworthy evidence and have transparently reported these methods here for the wider rapid review and scientific community.

## Background

Rapid reviews have emerged as a streamlined approach to synthesizing evidence in a timely manner -typically for the purpose of informing emergent decisions faced by decision makers in health care settings. Although there appears to be a growing use of rapid review 'methods', and a proliferation of rapid review products, there is a dearth of published literature on rapid review methodology. With limited transparency, it is impossible to determine the validity, appropriateness and, ultimately, the utility of these products. To address the gap, this paper outlines our experience with rapidly producing, publishing and disseminating evidence summaries in the context of our Knowledge to Action (KTA) research program to date.

The KTA research program is a two-year project designed to develop and assess the impact of a regional knowledge infrastructure that supports evidence-informed decision making by regional managers and stakeholders. It is a collaborative effort between researchers with expertise in knowledge synthesis and knowledge translation based at the Ottawa Hospital Research Institute (OHRI), and knowledge users involved in decision making around health service delivery based at the Champlain Local Health Integration Network (LHIN) - a regional health authority in Ontario, Canada. As part of the knowledge intelligence services that comprise the knowledge infrastructure, we have developed 'evidence summaries', our form of rapid review, which have come to be a flagship component of this project.

According to the Cochrane handbook, a traditional systematic review is a review that "attempts to collate all empirical evidence that fits pre-specified eligibility criteria in order to answer a specific research question. It uses explicit, systematic methods that are selected with a view to minimizing bias, thus providing more reliable findings from which conclusions can be drawn and decisions made" [1]. While systematic reviews are considered to be the gold standard in knowledge synthesis, they are not without their limitations. For example, they usually require between 6 months and 2 years to complete and often focus on a narrow clinical question or set of questions (see Table 1 for a comparison of rapid review versus systematic review). Policymakers, decision makers, stakeholders and other knowledge users, however, often require access to contextualized resources that succinctly and methodically address a broader scope of scientific evidence quickly [2]. Rapid review is an emerging methodology (or possible set of methodologies) within the broader knowledge syntheses repertoire that has evolved to address this need. A growing number of organizations, across various jurisdictions and countries, appear to be experimenting with this approach [3].

**Table 1:** General comparison of rapid review versus systematic review approaches ^a^

	Rapid review	Systematic review
**Timeframe**^**b**^	≤ 5 weeks	6 months to 2 years
**Question**	Question specified *a priori *(may include broad PICOS)	Often a focused clinical question (focused PICOS)
**Sources and searches**	Sources may be limited but sources/strategies made explicit	Comprehensive sources searched and explicit strategies
**Selection**	Criterion-based; uniformly applied	Criterion-based
**Appraisal**	Rigorous; critical appraisal (SRs only)	Rigorous; critical appraisal
**Synthesis**	Descriptive summary/categorization of the data	Qualitative summary +/- meta-analysis
**Inferences**	Limited/cautious interpretation of the findings	Evidence-based

**^a^**Specific to the KTA program - other groups have experimented with other approaches of rapid review and will therefore have other differences; ^b^Primary difference; other potentially important differences are noted in the cells. PICOS = population, interventions, comparators, outcomes and study designs; SR = systematic review.

Despite the increasing production and use of rapid review products, its methodology remains underdeveloped. In fact, there is no universally accepted definition of what constitutes a rapid review. Given their potential deficits in the absence of an approved methodology, many experts have questioned the validity of rapid reviews [3,4]. Given this lack of definition and evolving landscape, we have abstained from applying the label 'rapid review' to our KTA syntheses, and have alternatively called them 'evidence summaries'. Despite this, we consider our evidence summaries to be part of the continuum of rapid reviews, as conceptualized by Ganann and colleagues [3] and thus, in developing our evidence summary methods, we aim to advance to the understanding of rapid reviews as a whole.

### General approach

The KTA evidence summary was conceived as an overview of the available evidence addressing a research question or set of research questions related to a single topic (an area of need and priority identified by knowledge users of the Champlain LHIN) produced within a short timeframe (four to five weeks). Because they were developed under the auspices of the KTA research program, and therefore as a means to facilitate collaboration between researchers of the OHRI and knowledge users of the Champlain LHIN, the first several evidence summaries were developed in the absence of a specific methodology; rather, their production was informed by a general intent to consult and synthesize a broad range of quality evidence quickly.

Following positive responses to early evidence summaries and the progressive development of a collaborative relationship between the OHRI and Champlain LHIN, considerable effort had been invested toward the advancement of their methods. For example, we have continued to experiment with incorporating more of the established methods of systematic reviews, while maintaining our capacity to deliver a final product in a timely manner. We have also experimented with various user-friendly formats, reporting styles and document lengths to increase usability. Usability has been an oft-neglected component in traditional systematic reviews despite evidence that has shown its capacity to improve the quality and cost effectiveness of health care [5,6].

Potential uses of an evidence summary may include the following:

• to serve as an informative brief that prepares stakeholders for discussion on a policy issue;

• to support the direction and evidence-base for various health policy initiatives;

• to support the development of clinical interventions and/or health services programs.

From November 2009 to March 2011 we have produced 11 evidence summaries. Our eight-step approach for producing evidence summaries has been developed iteratively; it is outlined in Table 2 and described below.

**Table 2:** Outline of eight steps informing Knowledge to Action evidence summary approach

Knowledge to Action step	Task
**Step 1**	Needs assessment
**Step 2**	Question development and refinement
**Step 3**	Proposal development and approval
**Step 4**	Systematic literature search
**Step 5**	Screening and selection of studies
**Step 6**	Narrative synthesis of included studies (including assignment of evidence level)
**Step 7**	Report production
**Step 8**	Ongoing follow-up and dialogue with knowledge users

## Evidence summary methods

### Needs assessment

Our evidence summaries begin with a proposal for an evidence synthesis by a knowledge user about a clinical or health services or system topic. We have defined knowledge users in the context of the Champlain LHIN as policymakers, administrators, stakeholders, managers or decision makers. Probing consultation with knowledge users is recognized as an important initial stage of synthesizing information for the purpose of supporting evidence-informed decision making [7]; as such, the needs assessment phase has formed the cornerstone of our evidence summaries from the beginning. The purpose of these consultations is to ascertain the scope of the question to be addressed, the purpose for which the evidence summary will be used and the availability and commitment of the knowledge user for continued collaboration during the project period.

In particular, we have found that the research team may need to elicit additional information from knowledge users about the specific needs and interests related to their proposed topic or question. This process serves the dual objective of refining the scope such that it is suitable to our proposed methods and ensuring that the final product is meaningful and useful for its intended audience and their objectives. In cases where a proposed topic has been difficult to address in the context of a rapid evidence summary (for example, interest in the risk factor of a condition rather than the efficacy of an intervention to address it), we have strived to retain the knowledge users' priorities and objectives and, where feasible and appropriate, adapt our methods to address their specific needs. Our methods and evidence summaries have benefited from this dynamic approach.

### Question development and refinement

Generally, knowledge users in our context have not demonstrated a strong capacity for formulating effective research questions; while they are clear about the broad strokes of what they want to ask, they seem less able to provide insight into the critical details that make a research question more precise and, therefore, answerable. To address this problem, we have established a requirement that knowledge users make an initial time investment (around 1 to 2 hours in addition to the needs assessment) to collaboratively develop a clear and effective research question. Using information gleaned from the initial needs assessment (and possibly an environmental scan of the literature), the research team attempts to facilitate the question refinement process by proposing research questions to be vetted by the knowledge user. For questions of effectiveness, we attempt to operationalize the Participants/Populations, Interventions, Comparators, and Outcomes framework as reasonably as possible; when addressing questions related to health systems and/or health services, we modify accordingly. This is an iterative process whereby questions are edited and refined by both the research team and knowledge user until a suitable research question (or series of questions) satisfying both parties (as outlined in the needs assessment section above) has been established. Questions posed by evidence summaries to-date can be accessed on the KTA website http://www.ohri.ca/kta and have included the following (in chronological order):

1. Pre-diabetes

2. Health services for the 21^st ^century

3. Electronic health records

4. What is known about postpartum interventions for women with a history of gestational diabetes mellitus?

5. What is known about the timing of elective repeat Cesarean section?

6. What is known about options and approaches to fetal surveillance and intrapartum management of women with gestational diabetes mellitus?

7. What evidence exists to describe the effect of interventions that use pedometers to reduce risk for and manage chronic disease?

8. What are the drivers of in-hospital formula supplementation in healthy term neonates and what is the effectiveness of hospital-based interventions designed to reduce formula supplementation?

9. What is known about third and fourth degree lacerations during vaginal birth?

10. What is known about the maternal and newborn risks of non-indicated induction of pregnant women at term?

11. What is the evidence of the effectiveness and safety of emergency department short stay units?

12. Of note, the first three evidence summaries were produced in the absence of a primary research question but rather using a series of research questions developed by the research team. In part, the lack of clarity and direction supporting these efforts was a primary precipitating factor behind the researchers' subsequent demand for greater involvement of the knowledge users in formulating and establishing precise research questions.

### Proposal development and approval

In the initial stages of the KTA project, it was not yet clear to the research team which of the program's offerings would be most useful to Champlain LHIN knowledge users. As such, the first few evidence summaries primarily reflected an effort on the part of the research team to identify what may or may not be useful for the knowledge users of the Champlain LHIN. Because of this, a formal proposal was not produced for these first few evidence summaries. However, as enthusiasm among knowledge users grew and additional evidence summaries were requested, it became clear that there was a need for a formal document to succinctly summarize the outcomes of the needs assessment and question refinement stages as well communicate any other particulars relevant to the completion of the report that may or may not have been discussed. In its current form, the proposal aims to include: question background; finalized research question or questions; proposed methods, deliverables and timelines; and knowledge user-research team agreements (for example, availability of knowledge users during project). In addition to providing a point of reference for the knowledge users and research team (and allowing identification of possible misinterpretations) the proposal may also serve to inform extended members of the research team (such as the information scientist) and provide necessary documentation for securing outside funding, when applicable. A brief and concise proposal template has been developed to expedite this step, thus maximizing the time for conducting the evidence summary itself.

### Systematic literature search

Depending on the nature of the question, purpose of the report and magnitude of the literature, a variety of types of evidence have been targeted in the searches for evidence summaries. In most cases (for example, for questions of treatment effectiveness), emphasis has been placed on locating and summarizing evidence from relevant and high quality systematic reviews. Evidence from systematic reviews is prioritized in order to limit unnecessary duplication, to minimize resources needed to screen and summarize primary level evidence and to minimize the potential bias and/or error which could be incurred by reviewing primary evidence rapidly. In the absence of systematic reviews, however, high-quality and/or recent primary studies may be included, as well as landmark and/or oft-cited studies. Commentaries may also be considered (although rarely) as a means of providing background and/or context to the body of literature, as well as guidelines, economic analyses and other non-clinical reports. Finally, quasi-experimental and/or observational studies of high quality (such as prospective, rigorous quantitative analyses) may also be considered. The inclusion of evidence from primary studies and other reports was more commonly employed with earlier summaries, when our rigorous approach was still being refined and the questions being addressed were broader and more topic-based. Selection of 'high-quality' studies was determined subjectively, based on methodological expertise, by the research coordinator responsible for the particular evidence summary. Recent summaries have addressed narrower questions and, with the exception of relevant information garnered from guidelines, have almost exclusively drawn from evidence reported in systematic reviews. All literature is considered regardless of publication status.

Searches for the first four evidence summaries were conducted by the research coordinator in the absence of an information specialist, any reference management or study selection software, or the use of tools to search for grey literature. As enthusiasm grew among knowledge users, we aimed to improve upon the rigor of our methods by consulting a senior information scientist to perform systematic searches, ensuring appropriate concepts, keywords and subject heading terms, as well as relevant grey literature, were incorporated. For example, to ensure comprehensive coverage of grey literature, the information scientist uses *Grey Matters*, a seminal guide for searching grey literature produced by the Canadian Agency for Drugs and Technologies in Health [8]. With the exception of one evidence summary, all subsequent evidence summaries have incorporated this expertise.

To manage the records retrieved, search yields are downloaded into a bibliographic database software (Reference Manager^®^; Thomson Reuters, New York, NY) where search strategies, dates, yield and duplicate counts are recorded in a search log. Citations are then uploaded to DistillerSR^© ^(Evidence Partners Inc. Ottawa, Canada), an internet-based systematic review software program intended to facilitate study selection by the research team.

### Screening and selection of studies

Once citations have been uploaded to DistillerSR^©^, the research coordinator establishes levels for screening citations (such as title, abstract, full text), enters screening questions within these levels and sets limits and rules regarding the screening process (for example, the number of reviewers needed to include and exclude citations). The screening questions operationalize the eligibility criteria; initially these were informed by the needs assessment, however, subsequent to the addition of the question refinement stage, they were informed by the study question.

Usually the retrieval of full text documents takes place following one level of title and abstract screening. Occasionally, however, upon first pass of the literature, researchers will discuss and refine eligibility criteria with the knowledge users and, using the revised criteria, a second level of screening title and abstracts is performed. Full text is obtained largely through the journal subscriptions held by the Ottawa Hospital and the University of Ottawa; records not available electronically are pragmatically excluded as timelines do not permit for delays incurred by interlibrary loans. Similarly, due to limited time and resources available for translation, only English reports are included. Although exclusion of languages other than English is undesirable, it is considered to be a reasonable practice given limited time and resources and there is some evidence to suggest that it may not markedly bias review findings [9]. In most cases, the research coordinator will perform one or two final rounds of screening of the full texts before arriving at the final set of records to be synthesized.

Screening for the first few evidence summaries was undertaken by one reviewer. It is recognized, however, that a single reviewer introduces a level of error that is not desirable [10], and a second reviewer is now generally included on all evidence summaries, typically to review records excluded by the first reviewer - an approach we have labeled 'liberal accelerated'. For our most recent evidence summary, two reviewers (one with methodological expertise, one with clinical expertise) independently reviewed all records. Due to time constraints, however, conflict could only be resolved by passing conflicted records up to the next level of screening rather than through consensus, and ultimately the final decision as to which reports were summarized was made by the research coordinator preparing the report.

### Narrative synthesis of included studies (including risk-of-bias assessment)

While about half of published systematic reviews include a meta-analysis [11], the KTA evidence summaries do not undertake this level of quantitative synthesis (although we do report the results of included meta-analyses). Rather, the final report is designed to provide an overview of the evidence identified, organized in an intuitive way, with the goal of providing knowledge users with a sense of the volume and direction of available evidence addressing the topic of interest. As such, evidence summaries are typically produced by extracting the primary objective, methods, results and relevant limitations from each included systematic review or primary study. When including evidence from cost-effectiveness studies, recommendations from guidelines or other included reports, only the most relevant material (determined subjectively by the reviewer) is extracted and presented. Thus far, this task has been carried out by the projects' research coordinator.

In addition to summarizing the evidence, each study is assigned a level of evidence based on a modified framework established by the Cochrane Musculoskeletal Group [12]. The goal of using this simple hierarchal grading system is to allow knowledge users to make a cursory determination as to whether a study has a higher or lower risk of bias, based on the design of the study itself (for example, systematic reviews are rated higher than randomized controlled trials, which are in turn rated higher than observational studies and expert opinion). Particular care is taken in the reporting of evidence from non-systematic reviews, such that only the highest quality reports are summarized and limitations and potential biases are disclosed. For example, if observational studies are included to answer a question about risk factors for an outcome, we may only select studies in which data has been collected prospectively and on which a rigorous quantitative analysis has been performed (such as multivariable logistic regression); further, caution would be encouraged in interpreting these findings. Recent evidence summaries have also employed the 'assessment of multiple systematic reviews' (AMSTAR) tool as a means of assessing the methodological quality of included systematic reviews [13]. Both the level of evidence and AMSTAR score are presented at the beginning of each record summarized. We continue to consider ways of refining assessments of, and presenting, the risk of bias to knowledge users.

### Report production

**Figure 1:**
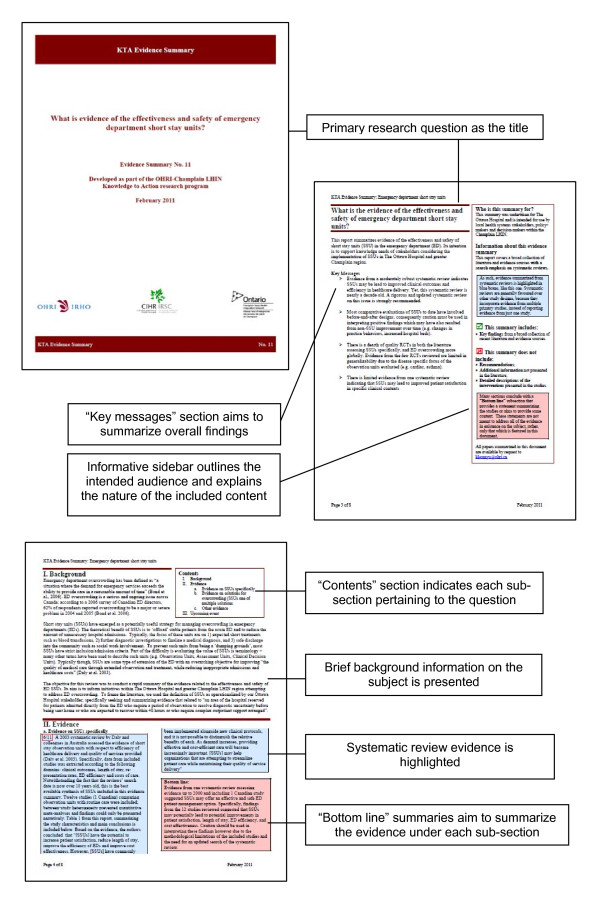
**Short stay unit evidence summary (excerpt)**.

This step includes developing a concise report that succinctly yet methodically covers all components we set out to address in the proposal. An initial template for the evidence summary report was developed iteratively based on early feedback from the knowledge users. Later formats have incorporated ideas from the structured summaries of systematic reviews developed by the Supporting Policy relevant Reviews and Trials Collaboration [14] as well as evidence reviews produced by the Centre for Clinical Effectiveness [15], in addition to continued feedback from knowledge users and brainstorming from the research team. The evidence summary template now includes a cover page introducing the question and partnerships, a summary page highlighting the key messages of the report and describing its intended audience, a disclosure page, a page that outlines the background to the question and a table of contents, the body of the report that summarizes included studies and provides bottom line statements, a reference list, a methods page, and a final information page including acknowledgements and author information (Figure 1). Most evidence summaries are between 10 and 15 pages long.

Once the content of the report has been inserted, the final phase of aesthetic formatting occurs (for example, arranging text blocks and wrapping text in a visually-pleasing manner, inserting page breaks where necessary, formatting headers, footers and page numbers). We have learned not to underestimate the time needed to perform this critical last step which, in addition to the quality of the content, we believe may facilitate dissemination and implementation of the evidence summaries to its intended audience.

### Ongoing follow-up and dialogue with knowledge users

Evidence summaries were conceived within the context of a research program that seeks to build relationships between health policymakers and health services researchers. As such, the evidence summaries developed by the research program serve as a basis for researchers to learn more about what health knowledge users need to incorporate evidence into their work. We continue to engage with knowledge users (see Case Example in Table 3), both informally and formally, regarding the utility of evidence summaries for meeting their decision and policy making needs. In some instances, this feedback has led to the revision of an evidence summary to better address their needs, whereas at other times it has served to inform the evolution of our methods. Overall, a collaborative approach appears to have a mutually beneficial effect for both Champlain LHIN knowledge users and OHRI researchers. Formal evaluation through the use of key informant interviews is ongoing.

**Table 3:** Case example of a recent evidence summary

Broad components	Specific details
**Context**	Acute care hospital (The Ottawa Hospital) TQH
**Problem**	Overcrowding in the emergency room
**Knowledge users**	Senior hospital management
**Question of interest**	What is the safety and effectiveness of short stay units in the emergency department?
**Evidence summary process**	• The principal knowledge user was integrally involved in Steps 1 through 3 (Needs assessment; Question development and refinement; Proposal development and approval) • A clinical expert provided key conceptual feedback during Steps 1 and 2 and contributed to the writing of the proposal (Step 3) • The information scientist developed and executed the search strategy (Step 4) • The clinical expert also served as the reviewer for Step 5, along with the research coordinator • Steps 6-7 were completed by the research coordinator; the final report was edited and approved by the principal knowledge user and clinical expert^a^ • The principal knowledge user distributed the evidence summary (Figure 1) to TOH senior hospital management and the wider clinical team; follow-up with knowledge users (Step 8) is ongoing.
**Outputs**	• Positive response to the evidence summary among both the clinical and senior management teams; the evidence summary is being used to inform discussions considering the implementation of a short stay unit • Development of a positive collaborative relationship between the research team and knowledge users has led to the decision to pursue a collaborative knowledge synthesis opportunity on this topic.

^a ^Available at http:\\www.ohri.ca/kta

## Discussion

As rapid review products, our evidence summaries inherently harbor limitations relative to systematic reviews in that they are produced within a short timeframe using limited resources. The methods do not employ as much rigor as would be applied in a traditional systematic review and evidence summaries may therefore be subject to a greater degree of bias and/or error. While research comparing rapid with systematic review is limited, it is worth noting that a 2008 study by Watt *et al*. found that, despite "axiomatic differences" between the rapid and full reviews evaluated, "the essential conclusions of the rapid and full reviews did not differ extensively" [4], suggesting that products like the KTA evidence summaries may offer a useful and valid approach.

The aim of our evidence summary approach is to deliver evidence in both a timely manner and usable format and thus there is tension between the rigor used to conduct an evidence summary and the timeline within which the information is required by the knowledge user. Many decision makers require the evidence emergently or urgently; waiting 6 months or more for a full systematic review is not an option. We currently have no clear evidence about the willingness of decision makers to compromise methodological rigor in order to get a quicker answer, although we are aware of at least two other rapid response services in Canada which offer a sliding scale of available rapid syntheses based on different lengths of time [16,17]. Perhaps most important is a clearer understanding of what effect is had by adapting the methods of systematic review into a short timeline and to what extent this practice is valid. For example, Parkill and colleagues have shown that, when compared to highly sensitive searches, pragmatically-based searches conducted for the purpose of populating an evidence map can produce a similar amount of relevant records, more quickly and at less cost [18]. Further research evaluating the relative impact of streamlining other systematic review methods is required.

Additionally, the purposes for and context within which rapid review products are used should be carefully considered when assessing their value [4]. For example, the KTA research program participants are policy and decision makers; preliminary outcome data suggests that these users often employ evidence summaries as background for multiple stakeholders from a wide variety of disciplines taking part in discussions that form the basis of development and/or implementation of health services initiatives. In this context, an overview of the evidence, with a systematic search component and user-friendly final report, may be considered reasonable and appropriate. That is to say, a modestly robust summary of the evidence is better for informing a health services decision than no evidence at all.

Finally, we continue to explore ways in which we can advance the methods of evidence summaries. An example is the issue of optimal page length for an evidence summary; tension exists between rigorous transparency and inclusivity and providing a concise and readable summary. Traditional systematic reviews vary in length but most are too long and technical to read for busy decision makers. Keeping evidence summaries short, under 10 pages, is likely important and has been confirmed by our knowledge users. In addition, while there is a need to be transparent regarding methods, there is an equally important need to promptly answer the question posed by the knowledge user. Increasingly, our evidence summaries have been very selective as to the methodological detail presented about summarized articles, and has moved the brief description of the methods informing the evidence summary itself to the back of the report. These changes have been the result of direct feedback from end users as well as general experimentation in our reporting approach. In addition, we have begun to explore incorporating a summary of findings table into the evidence summary in order to provide the knowledge user with the information they need as quickly as possible. The British Medical Journal has started experimenting in a similar manner with reports of randomized trials [19]. It is expected that a summary of findings table presents the evidence in a more succinct and user-friendly format. Another consideration is a Grades of Recommendation, Assessment, Development and Evaluation-like assessment for the overall body of information included in the evidence summary as an important feature for knowledge users' interpretation of the global evidence base [20]. Discussion with various decision makers and stakeholders around the importance and relevance of these proposed improvements is ongoing.

## Summary

Rapid review is an emerging approach within the world of knowledge synthesis for providing evidence to decision makers in a short timeframe. There are gaps in transparency and in knowledge about the trustworthiness of rapid reviews. We hope this paper serves to narrow those gaps, however it does not (and cannot be expected to) eliminate them completely. While research remains ongoing, our experience to-date has shown KTA evidence summaries to be effective tools for addressing the evidentiary needs of health services decision makers in the Champlain region and are highly valued by researchers and knowledge users alike.

## Competing interests

DM is a co-editor-in-chief at Systematic Reviews.

## Authors' contributions

SK contributed to the design, coordination and execution of the study and its associated evidence summaries and drafted the manuscript. KK contributed to the design, coordination and execution of the study and its associated evidence summaries and oversaw revisions of the manuscript. RC contributed to the conception, design and execution of the study. JG contributed to the conception, design, coordination and execution of the study. DM contributed to the conception, design, coordination and execution of the study and its associated evidence summaries. All authors participated in the critical revision and read and approved the final manuscript.
